# Comparison of anti-thymocyte globulin-based immunosuppressive therapy and allogeneic hematopoietic stem cell transplantation in patients with transfusion-dependent non-severe aplastic anaemia: a retrospective study from a single centre

**DOI:** 10.1080/07853890.2023.2271475

**Published:** 2023-10-23

**Authors:** Yingying Shen, Yuzhu Li, Qi Liu, Wenbin Liu, Qinghong Yu, Huijin Hu, Shan Liu, Jingjie Dong, Min Xu, Yaonan Hong, Ying Chen, Shu Deng, Haifeng Zhuang, Zhiping Hu, Shenyun Lin, Yiping Shen, Jianping Shen, Yuhong Zhou, Baodong Ye, Dijiong Wu

**Affiliations:** a Department of Hematology, The First Affiliated Hospital of Zhejiang Chinese Medical University (Zhejiang Provincial Hospital of Chinese Medicine), Hangzhou, Zhejiang, China; b The First School of Clinical Medicine, Zhejiang Chinese Medical University, Hangzhou, Zhejiang, China; c Department of Clinical Evaluation Center, The First Affiliated Hospital of Zhejiang Chinese Medical University (Zhejiang Provincial Hospital of Chinese Medicine), Hangzhou, Zhejiang, China; d Department of Hospital Administration, The First Affiliated Hospital of Zhejiang Chinese Medical University, Hangzhou, Zhejiang, China

**Keywords:** Hematopoietic stem cell transplantation, immunosuppressive therapy, transfusion-dependent, non-severe aplastic anaemia

## Abstract

**Objectives:**

The selection and timing of anti-thymocyte globulin (ATG)-based immunosuppressive therapy (IST) or allogeneic hematopoietic stem cell transplantation (allo-HSCT) in patients with transfusion-dependent non-severe aplastic anemia (TD-NSAA) pose significant clinical challenges. This study aims to compare the efficacy and long-term outcomes of the two treatments in TD-NSAA.

**Methods:**

Patients who underwent ATG-based IST or allo-HSCT between July 2011 and December 2019 were reviewed. We gathered their clinical information, treatment response, survival data, and subsequently analysed the associated risk factors.

**Results:**

A total of 97 TD-NSAA patients were reviewed, and 55 patients who underwent either ATG-based IST (*n* = 27) or allo-HSCT (*n* = 28) were enrolled. We observed a significant disparity in the 12-month overall response rate (ORR) (48.1% in IST vs 78.6% in HSCT, *p* < 0.05), but not in five-year overall survival (OS) and event-free survival (EFS). Multivariate Cox regression analysis identified the transfusion of ≥78.75 units of red blood cells (RBCs) as the sole independent risk factor for OS (HR: 17.04, *p* = 0.039) in the IST group. For the HSCT group, disease duration (DD) ≥20 months and transfusion of ≥78.75 units of RBCs predicted an adverse EFS. Frontline IST exhibited superior 12-month ORR (68.8% vs 18.2%, *p* = 0.018) and five-year EFS when compared to non-frontline. Patients with a DD ranging from 6 to 20 months displayed a better EFS (*p* = 0.016) in HSCT group than those in the ATG-based IST group.

**Conclusions:**

Prior treatment history, disease duration, and serum ferritin levels should be carefully weighed when making the choice between ATG-based IST and allo-HSCT for TD-NSAA.

## KEY MESSAGES

1. The selection and timing of anti-thymocyte globulin (ATG)-based immunosuppressive therapy (IST) or allogeneic hematopoietic stem cell transplantation (allo-HSCT) present notable clinical challenges for individuals with transfusion-dependent non-severe aplastic anaemia (TD-NSAA).
2. In terms of treatment outcomes, allo-HSCT exhibited a higher 12-month overall response rate (ORR) in comparison to ATG-based IST among TD-NSAA patients. Nevertheless, comparable rates of 5-year overall survival (OS) and event-free survival (EFS) were observed between the two therapeutic approaches.
3. Several factors warrant consideration when deliberating between ATG-based IST and allo-HSCT for TD-NSAA. These factors include the patient’s prior treatment history, disease duration, number of packed red cell transfusions received, and serum ferritin levels.

## Introduction

Aplastic anaemia (AA) constitutes a rare hematologic disorder characterized by a scarcity of bone marrow cells and the presence of pancytopenia [1]. The severity of AA can be classified into severe aplastic anaemia (SAA), very severe AA, and non-severe aplastic anaemia (NSAA), based on the extent of bone marrow cellularity and cytopenia [1]. Transfusion-dependent non-severe aplastic anaemia (TD-NSAA) encompasses cases of AA that necessitate blood transfusions, yet do not meet the criteria for SAA. Observations of its natural history have underscored that the progression-free survival (PFS) of transfusion-independent NSAA substantially diminishes, from approximately 62% at 60 months post-diagnosis to 22% at 120 months [2]. While allogeneic hematopoietic stem cell transplantation (HSCT) and anti-thymocyte globulin (ATG)-based immunosuppressive therapy (IST) stand as the foremost treatments for SAA, their application is not advocated for TD-NSAA [3]. In China, the pragmatic guideline recommends initiating treatment with cyclosporine (CsA)-based IST, sometimes coupled with hematopoietic-stimulating protocols like androgens. If no response (NR) is observed within 6 months, further treatment options are considered, possibly escalating to treatment strategies suitable for SAA [4]. It has been documented that the hematopoietic response elicited by frontline ATG-based IST (involving ATG combined with CsA) surpasses that of solitary CsA-based IST (75% vs. 55.8% after 6 months) for TD-NSAA. However, relapse and the development of clonal expansion (resulting in myelodysplastic syndrome [MDS] or acute myeloid leukaemia [AML] transformation) remain inevitable occurrences [5]. Notably, HSCT demonstrates superior long-term PFS compared to ATG-based IST, though its implementation is restricted due to potential complications such as graft dysfunction, graft-versus-host disease, diverse infections, and transplantation-related mortality, alongside the challenges posed by donor availability [6]. For paediatric TD-NSAA, HSCT is considered a practical, effective, and [7] secure therapeutic avenue [7]. Presently, limited data are available for the comparative assessment of ATG-based IST and HSCT efficacy in TD-NSAA. With the advent of haploidentical transplantation in China, a more suitable donor pool has become available for a substantial portion of TD-NSAA patients. In this retrospective analysis, we aim to scrutinize the effectiveness of ATG-based IST and HSCT in TD-NSAA patients and identify potential prognostic risk factors.

## Patients and methods

### Study design

We conducted a thorough review of 97 patients diagnosed with TD-NSAA at The First Affiliated Hospital of Zhejiang Chinese Medical University between July 2011 and December 2019. Exclusion criteria encompassed patients who exclusively received supportive treatment (*n* = 5), sole CsA treatment (*n* = 30), and individuals outside the age bracket of 12 to 60 years (*n* = 7). Consequently, 55 patients qualified for the comparative evaluation of treatment effectiveness and prognostic outcomes between ATG-based IST and HSCT (refer to Supplemental Figure 1). Pertinent baseline characteristics, such as age, gender, disease duration (DD), transfusion dependency, blood routine parameters, ferritin levels, and T-lymphocyte subsets (including CD3 + CD4+ T helper cells, CD3 + CD8+ cytotoxic T cells, CD4+/CD8+ ratio, and CD4+/CD25+/CD127- regulatory T cells) before commencement of treatment, were meticulously documented. Additionally, treatment response rates and survival metrics, specifically overall survival (OS) and event-free survival (EFS), were meticulously compared. Comprehensive written or oral informed consent for participation was obtained from both patients and donors. This study has been registered at chictr.org.cn under # ChiCTR2200065804.

### Patients

Enrolled patients adhered to the complete set of the following criteria, as per the guidelines of the British Society for Standards in Haematology concerning AA [3]: (1) presence of bone marrow hypoproliferation at multiple sites (≥2, including both sides of the posterior superior iliac spine and sternal manubrium in severe cases); (2) diagnosis of NSAA consistent with the Camitta criterion [8]; (3) documented transfusion dependency, with platelet (PLT) counts <10 × 10^9^/L and/or hemoglobin (Hb) levels <60 g/L; (4) receipt of either ATG-based IST or HSCT treatment; and (5) age falling within the range of 12 to 60 years. Further, a rigorous diagnostic approach involving bone marrow analyses, flow cytometry, next-generation sequencing, and immunological assessments was employed to exclude other pancytopenia disorders such as congenital AA, MDS, paroxysmal nocturnal hemoglobinuria (PNH), and immune-related cytopenia.

### Treatment regimens

Patients within the ATG-based IST group were administered rabbit ATG (thymoglobulin, Sanofi, France) at a dose of 3 mg/(kg ⋅ day) for five consecutive days. Additionally, methylprednisolone was intravenously administered at a dose of 1 mg/(kg ⋅ day) from day 1 to day 15 to forestall serum sickness reaction to rabbit ATG. Subsequently, a tapering dose was administered until discontinuation on day 28. Oral CsA was initiated on day 1 at a dose of 5 mg/(kg ⋅ day), divided into multiple doses, and tailored to maintain a whole blood concentration ranging from 150-250 ng/mL until the optimal response was achieved. Patients belonging to the HSCT group underwent FLU/CY/ATG (fludarabine 150 mg/m^2^ in total, cyclophosphamide 120-180 mg/kg in total, and r-ATG 7.5-10 mg/kg in total)- or BU/CY (busulfan 6.4 mg/kg in total and cyclophosphamide 200 mg/kg in total)-based conditioning regimens. The choices of conditioning regimen were based on the degree of bone marrow failure, most HSCT patients experienced FLU/CY/ATG regimes, regardless of the sources of the donor. Peripheral blood stem cells were harvested, either with or without bone marrow, following mobilization with granulocyte colony-stimulating factor (G-CSF). The GVHD prophylactic regimen encompassed intravenous cyclosporin A (CsA), short-term intravenous methotrexate (three dosages, on day +1, +3 and +6 respectively), and oral mycophenolate mofetil. Administration of G-CSF, erythropoietin, recombinant human thrombopoietin, thrombopoietin receptor agonists, and blood transfusion were permissible prior to successful stem cell engraftment.

### Donor selection

Donor categorization was determined through human leukocyte antigen (HLA) matching, encompassing HLA-identical sibling donors, unrelated donors (UDs), and other family donors. In our cohort, UDs were mandated to exhibit a 10/10 or 9/10 HLA match for class I (HLA-A, -B, -C) and class II (HLA-DRB1, -DQB1) antigens [3]. A fully matched unrelated donor (MUD) was precisely defined as a 10/10 match for HLA class I and class II antigens [9]. An alternative donor (AD) alluded to a haploidentical family donor or a 9/10 MUD. A matched donor incorporated matched sibling donors (MSDs) or MUDs. Within this study, MSDs were the preferred choice for HSCT in TD-NSAA patients. MUDs were evaluated for patients lacking an available MSD and who were under the age of 50 years. Notably, haploidentical HSCT (AD transplantation) was conducted more frequently at our centre compared to fully matched UD or one-allele mismatched UD.

### Definitions and evaluations

The point of initiation for the study was precisely delineated as the inaugural day of ATG infusion in the ATG-based IST group or the reinfusion of hematopoietic stem cells in the HSCT group. The term ‘disease duration’ (DD) explicitly denoted the temporal interval between the diagnosis of TD-NSAA and the commencement of HSCT or ATG-based IST. ‘Frontline therapy’ was defined as individuals receiving less than 3 months of CsA-based IST prior to their inclusion in the study, whereas ‘non-frontline therapy’ pertained to patients who had previously undergone ATG-based IST or received at least 3 months of solitary CsA treatment before being enrolled in this study.

#### Response

Response evaluation was conducted at the 3rd, 6th, and 12th months following treatment initiation. Complete response (CR) was defined as a haemoglobin (Hb) level of ≥100 g/L and a platelet (PLT) count of ≥100 × 10^9^/L. Partial response (PR) was characterized by no longer requiring transfusions with an Hb level of ≥70 g/L and a PLT count of ≥30 × 10^9^/L. Patients who remained transfusion-dependent were categorized as non-response (NR). The overall response rate (ORR) was calculated by summing the rates of CR and PR. Progression referred to the development of severe aplastic anaemia (SAA) during re-evaluation. According to the literature [10], primary graft failure in this study was defined as the absolute neutrophil count (ANC) or PLT counts that still did not meet the standard for hematopoietic reconstitution on Day 28 after hematopoietic stem cell reinfusion. Secondary graft failure pertains to the recurrence of persistent ANC <0.5 × 10^9^/L and PLT < 20 × 10^9^/L after hematopoietic reconstitution, with loss of donor chimerism, or when the chimerism rate of donor cells in bone marrow without recurrence is <5%.

#### Survival outcome

To evaluate the survival outcomes of ATG-based IST and HSCT, the 5-year overall survival (OS) and five-year event-free survival (EFS) were estimated. The 5-year OS was the time from treatment initiation to the five-year follow-up or death. Events for EFS included death, disease progression, relapse, clonal expansion (transition to PNH/MDS/AML), NR within 12 months, or the necessity for conversion treatment in the ATG-based IST group. Furthermore, graft failure, post-transplant lymphoproliferative disorders (PTLD), or grade III–IV acute graft-versus-host disease (GVHD) were also considered as events for EFS in the HSCT group. Early mortality was defined as death within 100 days after HSCT or ATG-based IST treatment.

#### Multivariate survival risk analysis

The multivariate analysis encompassed treatment regimens, disease duration (DD), transfusion dependence, duration of previous treatment, age, pre-treatment blood cell count, serum ferritin (SF) level, number of transfused packed red cells (PRCs), and CD4/CD8 ratio, to assess independent risk factors for 5-year OS and EFS. ANC, Hb, PLT, reticulocyte (Ret), lymphocyte percentage, and CD4/CD8 ratio were grouped based on the median values. The number of transfused PRCs and DD were grouped using receiver-operating characteristic (ROC) curve outcomes (Supplemental Figure 2). Age was categorized according to the AA guideline [3]. The SF cut-off point was set at 1000 ng/mL [11]. The specific groupings were as follows: age <35 years, age 35-50 years, or age >50 years; DD <6 months or DD ≥6 months in the ATG-based IST group (Supplemental Figure 2 A); DD <20 months or DD ≥20 months in the HSCT group (Supplemental Fig. 2B); frontline therapy or non-frontline therapy; ANC <1 × 109/L or ANC ≥1 × 10^9^/L; Hb <54 g/L or Hb ≥54 g/L; PLT <11 × 10^9^/L or PLT ≥11 × 10^9^/L; Ret <35 × 10^9^/L or Ret ≥35 × 10^9^/L; SF <1000 ng/mL or SF ≥1000 ng/mL; number of transfused PRCs <78.75 U or ≥78.75 U (Supplemental Figure 2C); lymphocyte percentage <60% or lymphocyte percentage ≥60%; and CD4/CD8 ratio <1.10 or CD4/CD8 ratio ≥1.10.

### Complications

Acute graft-versus-host disease (aGVHD) was defined according to the Mount Sinai Acute GVHD International Consortium (MAGIC) criteria [12], while chronic graft-versus-host disease (cGVHD) was defined based on the National Institutes of Health Consensus Guidelines [13]. The incidence rates of aGVHD and cGVHD were compared among different graft types. Serum sickness reactions following ATG treatment (manifesting as fever, rash, joint swelling and pain, myalgia, etc.) [4], and infections within 3 months after treatment were documented.

### Statistical analysis

Statistical analyses were executed using SPSS 26.0. Patient characteristics were compared using the chi-square test for categorical variables and the Student *t*-test and non-parametric test for continuous variables. Hematologic responses were assessed using the chi-square test. Differences in 5-year OS and EFS between the ATG-based IST and HSCT groups were estimated using the Kaplan–Meier method with the log-rank test. Cumulative incidence of GVHD, cumulative probability of remission, and the probabilities of 5-year OS and EFS were calculated from the time of treatment initiation using the Kaplan–Meier method and compared between different groups using the log-rank test. The Cox proportional hazard model was employed to evaluate the multivariate effects on OS and EFS. A significance level of *p* < 0.05 was considered statistically significant.

## Results

### Baseline characteristics

In total, 55 patients (27 males, 49.09%) were included in this analysis, with a median age of 28 years (range: 16-60). Among them, 27 patients received ATG-based IST, while the remaining patients underwent HSCT. The baseline characteristics, including age, peripheral blood count, serum ferritin (SF), number of transfused packed red cells (PRCs), disease duration (DD), and lymphocyte subsets, were comparable between the two groups. However, the HSCT group had a significantly longer DD compared to the ATG-based IST group [12.5 months (range: 1–108) vs. 3 months (range: 1–73), *p* = 0.008]. Furthermore, the ATG-based IST group had a higher proportion of patients receiving frontline therapy (16/27 vs. 6/28, *p* = 0.006) and a shorter duration of previous treatment (*p* = 0.016) compared to the HSCT group (details displayed in Table 1). Within the HSCT group, patients received bone marrow or peripheral stem cells from donors with a median age of 32 years (range: 13-54). The donors included matched sibling donors (*n* = 8), matched unrelated donors (*n* = 3), and alternative donors (*n* = 17). Among the alternative donors, four were 9/10-matched unrelated donors, and 13 were haploidentical donors. Different conditioning regimens were employed, with two patients receiving BU/CY, two receiving BU/CY/ATG, and the remaining receiving FLU/CY/ATG-based regimens. Most patients received both peripheral blood and bone marrow stem cells (21/28, 75%), while the remaining received only peripheral blood (7/28, 25%). The absolute CD34+ cell count ranged from 3.50 × 10^6^/kg to 8.83 × 10^6^/kg, and the mononuclear cell count ranged from 3.56 × 10^8^/kg to 14.58 × 10^8^/kg (details outlined in Supplemental Table 1).

**Table 1.** Baseline characteristics of TD-NSAA patients.

	ATG based IST (*n* = 27)	HSCT (*n* = 28)	*P*
Age/ Median (range)	26 (16–60)	28.5 (16–50)	0.663
Age group/ *n*(%)			
<35 yr	19 (70.4%)	20 (71.4%)	
35–50 yr	5 (18.5%)	7 (25%)	
>50 yr	3 (11.1%)	1 (3.6%)	0.499
Gender/Male (%)	16 (59.3)	11 (39.3)	0.200
ANC(×10^9^/L)/Median (range)	0.9 (0.29–6.20)	1.0 (0.20–2.0)	0.360
Hb(g/L)/Median (range)	54 (35–71)	54.5 (32–99)	0.287
PLT(×10^9^/L)/Median (range)	11 (3–34)	10.5 (5–32)	0.923
Ret(×10^9^/L)/Median (range)	31.75 (11.32–80.14)	39.51 (11.93–64.94)	0.864
SF (ng/ml)/Median (range)	742.0 (95.3–4639.6)	756.3 (47.0–7991.5)	0.728
Serum immune globulin (g/L)/Median (range)			
IgA	1.63 (0.51–11.50)	1.71 (0.77–5.61)	0.641
IgG	9.07 (5.29–14.10)	10.34 (5.95–16.40)	0.162
IgM	1.12 (0.37–9.50)	1.51 (0.39–4.48)	0.912
CD4^+^CD25^+^CD127^-^ (% of CD4^+^CD25^+^)/Median (range)	4.3(2.1–8.1)	4.77 (1.6–10.7)	0.090
CD3^+^CD4^+^ (% of CD3^+^CD45^+^)/Median (range)	36 (6.67–55.44)	43.78 (18.12–57.24)	0.093
CD3^+^CD8^+^ (% of CD3^+^CD45^+^)/Median (range)	35 (15.07–58.24)	31.53 (20.2–65.93)	0.730
CD4^+^/CD8^+^ (ratio)/Median (range)	0.98 (0.42–2.28)	1.35 (0.29–2.37)	0.217
Disease duration (m)/Median (range)	3 (1–73)	12.5 (1–108)	***0.008***
Number of transfused PRCs (U)/Median (range)	16.5 (0–264)	72.5 (0–852)	0.080
Previous treatment	CsA ± androgens (20/27)	CsA ± androgens (27/28)	
	ATG ± CsA (2)	ATG ± CsA (3)	
	None (7)	None (1)	
Frontline	16	6	***0.006***
Non-frontline	11	22	
Duration of previous treatment			
<3 months/n	16	6	
3-6 months/n	0	0	
6-20 months/n	7	16	
>20 months/n	4	6	***0.016***
PNH clone/n (%)			
<1%	19 (67.9%)	15 (53.6%)	
1-10%	5 (18.5%)	10 (35.7%)	
10-20%	2 (7.4%)	3 (10.71)	
>20%	1 (3.7%)	0	0.325

Note: TD-NSAA: transfusion-dependent none-severe aplastic anemia; ANC: Absolute neutrophil count; Hb: hemoglobin; PLT: platelet; Ret: reticulocyte; SF: serum ferritin; PRCs: packed red cells; CsA: Cyclosporin A; ATG: antithymocyte globulin. Non-frontline therapy was defined as the patients had received ATG-based IST before enrolled, or had received CsA-based IST for more than 3 months. The paroxysmal nocturnal hemoglobinuria (PNH) clone was defined according to the percentage of glycosylphosphatidylinositol-deficient erythrocytes and/or neutrophils, as assessed by means of standard flow cytometry.

### Hematologic responses

**Figure 1.**
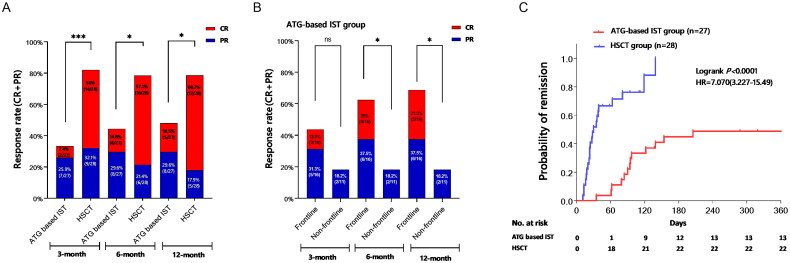
Comparison of clinical response between ATG-based IST group and HSCT group. 1A showed the Comparison of overall response rate (ORR, CR + PR) between ATG-based IST and HSCT 3, 6, and 12 months after treatment. 1B showed the ORR difference of the frontline and non-frontline therapy in ATG based IST group at different evaluation time points. 1C showed the time of achieving transfusion-free between groups. ns means no significant difference, ***<0.001, **<0.01, *<0.05.

All patients in the ATG-based IST and HSCT groups were eligible for efficacy analysis. The overall response rate (ORR) at the 3rd, 6th, and 12th months for patients receiving HSCT was 82.1% (including a 50% complete response, CR), 78.6% (with 57.1% CR), and 78.6% (with 60.7% CR), respectively. In contrast, the ATG-based IST group exhibited ORRs of 33.3% (7.4% CR), 44.4% (14.8% CR), and 48.1% (18.5% CR) at the corresponding time points (all *p* < 0.05, Figure 1(A)). Within the ATG-based IST group, patients who underwent frontline therapy showed an ORR of 62.5% (25% CR) at the 6th month, whereas the non-frontline therapy group displayed an ORR of 18.2% (with no CR) (*p* = 0.047). Similarly, at the 12th month, the frontline group exhibited an ORR of 68.8% (31.3% CR), compared to 18.2% (with no CR) in the non-frontline group (*p* = 0.018) (Figure 1(B)). The median time required to achieve transfusion independence was 24 days (ranging from 11 to 139) in the HSCT group and 94 days (ranging from 35 to 205) in the ATG-based IST group (*p* < 0.001, Figure 1(C)).

In terms of the effect of DD on clinical response, efficacy was evaluated after 12 months. Results indicated that patients with DD <20 months exhibited an ORR of 54.5% and 90.5% in the ATG-based IST and HSCT groups, respectively (*p* = 0.016). However, no significant difference was observed among patients with DD ≥20 months (20% vs. 42.9%, *p* = 0.575). Within the HSCT group, patients with DD <20 months displayed a higher ORR compared to those with DD ≥20 months (90.5% vs. 42.9%, *p* = 0.021). In contrast, no statistically significant differences were noted in the ATG-based IST group (*p* = 0.326).

### Evaluation of survival outcomes

#### Survival outcomes

**Figure 2.**
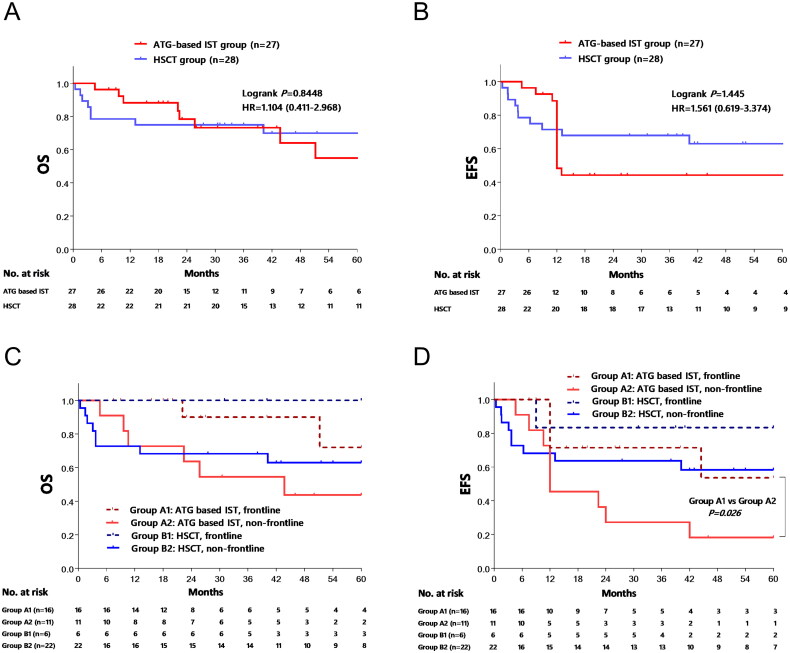
Comparison of estimated 5-year overall survival (OS) and event free survival (EFS). The OS (2A) and EFS (2B) of Entire population was compared, and no statistic differences were observed between ATG-based IST group and HSCT group. 2C and 2D showed the OS and EFS of different treatment regime based on first line or non-first line therapy by Kaplan–Meier method, respectively.

The median follow-up duration was 26 months (ranging from 5 to 123) in the ATG-based IST group and 41 months (ranging from 1 to 99) in the HSCT group. The 5-year overall survival (OS) rate stood at 56.5% ± 12.9% in the ATG-based IST group and 70.3% ± 8.9% in the HSCT group (*p* > 0.05) (Figure 2(A)). Likewise, the 5-year event-free survival (EFS) rate was 35.9% ± 11.4% in the ATG-based IST group and 63.3% ± 9.3% in the HSCT group (*p* > 0.05) (Figure 2(B)). Further analysis focusing on the timing of treatment in relation to survival indicated that the 5-year OS rate was 72% ± 17.8% in patients who received ATG-based IST as frontline therapy, compared to 43.6% ± 15.5% in the non-frontline therapy group (*p* = 0.059) (Figure 2(C)). The EFS rate was 53.6% ± 17.9% in the frontline group and 18.2% ± 11.6% in the non-frontline group (*p* = 0.026). However, no such difference was observed within the HSCT group (all *p* > 0.05, Figure 2(C,D)).

#### Univariate and multivariate COX analyses for survival risk factors

Both univariate and multivariate Cox proportional hazards regression analyses were conducted to determine independent prognostic factors influencing the prognosis of ATG-based IST or HSCT therapy. Patients were stratified into distinct subgroups based on treatment regimens, age, DD, duration of previous treatment, absolute neutrophil count (ANC), haemoglobin (Hb) level, platelet count (PLT), reticulocyte count (Ret), SF, number of transfused PRCs, lymphocyte percentage, and CD4/CD8 ratio. Univariate Cox regression analysis demonstrated that age, DD, SF, and the number of transfused PRCs exerted influence on the 5-year OS within the ATG-based IST group. Conversely, only the number of transfused PRCs yielded significance within the HSCT group (Table 2). Baseline variables deemed clinically relevant or displaying a univariate correlation with the outcome were included in a multivariate Cox proportional-hazards regression model. Given the significant correlation between DD and the number of transfused PRCs (*r* = 0.824, *p* < 0.001), the disease duration was excluded. Multivariate Cox analysis unveiled that a number of transfused PRCs ≥78.75 U constituted an independent risk factor for OS within the ATG-based IST group (HR: 17.04, 95% CI: 1.16-250.53, *p* = 0.039). Correspondingly, univariate analysis corroborated that the number of transfused PRCs and duration of previous treatment impacted the 5-year EFS in the ATG-based IST group. Moreover, patients with DD ≥20 months and a number of transfused PRCs ≥78.75 U were identified as predictors of unfavourable EFS in the HSCT group (Table 3). Furthermore, SF exhibited a substantial correlation with DD (*r* = 0.378, *p* = 0.004) and the number of transfused PRCs (*r* = 0.499, *p* < 0.001).

**Table 2.** Univariate analyses of factors affecting OS and EFS in the ATG based IST group.

Factors	OS			EFS		
	No. of events/ No. of patients	HR (95% CI)	*P*	No. of events/ No. of patients	HR (95% CI)	*P*
Age						
<35yr	5/19	Reference		11/19	Reference	
35–50yr	2/5	17.27 (1.45–205)	***0.024***	2/5	2.12 (0.43–10.37)	0.354
>50yr	1/3	1.08 (0.37–3.18)	0.883	1/3	0.70 (0.25–1.97)	0.497
Disease duration						
<6m	2/17	Reference		7/17	Reference	
≥6m	6/10	6.82 (1.33–34.86)	***0.021***	7/10	2.25 (0.88–7.84)	0.084
Duration of previous treatment						
<3 m ≥3m	2/16	Reference		5/16	Reference	
	6/11	4.19 (0.84–21.0)	0.081	9/11	2.99 (1.00–8.93)	***0.050***
Absolute neutrophil count						
<1 × 10^9/L	2/15	Reference		5/15	Reference	
≥1 × 10^9/L	6/12	4.74 (0.95–23.79)	0.059	9/12	1.96 (0.66–5.88)	0.229
Haemoglobin						
<54g/L	5/13	Reference		8/13	Reference	
≥54 g/L	3/14	0.36 (0.08–1.60)	0.181	6/14	0.50(0.17–1.46)	0.205
Platelet count						
<11 × 10^9/L	4/13	Reference		8/13	Reference	
≥11 × 10^9/L	4/14	1.16 (0.29–4.74)	0.832	6/14	0.71(0.25–2.08)	0.538
Reticulocyte count						
<35 × 10^9/L	6/17	Reference		11/17	Reference	
≥35 × 10^9/L	2/10	0.66(0.13–3.28)	0.609	3/10	0.45 (0.13–1.63)	0.227
Serum ferritin						
<1000ng/ml	2/16	Reference		6/16	Reference	
≥1000ng/ml	6/11	17.89(2.07–154.60)	***0.009***	8/11	2.77 (0.96–8.00)	0.060
Number of transfused PRCs						
<78.75U	2/19	Reference		7/19	Reference	
≥78.75U	6/8	22.56(2.63–193.65)	***0.004***	7/8	6.24(1.79–21.78)	***0.004***
Lymphocyte percentage						
<60%	5/15	Reference		8/15	Reference	
≥60%	3/12	0.53 (0.12–2.28)	0.395	6/12	1.06 (0.37–3.05)	0.918
CD4(+)/CD8(+) T cell						
<1.1	7/17	Reference		11/17	Reference	
≥1.1	1/10	0.21 (0.03–1.72)	0.146	3/10	0.38 (0.11–1.38)	0.141

**Table 3.** Univariate analyses of factors affecting OS and EFS in the HSCT group.

Factors	OS			EFS		
	No. of events/No. of patients	HR (95% CI)	*P*	No. of events/No. of patients	HR (95% CI)	*P*
Age						
<35yr	6/20	Reference		7/20	Reference	
35–50yr	2/7	0.81 (0.16–4.03)	0.799	3/7	1.08 (0.28–4.18)	0.913
>50yr	0/1	0.21 (0.0–528.1)	0.699	0/1	0.21 (0.0–265.2)	0.671
Disease duration						
<20m	4/21	Reference		5/21	Reference	
≥20m	4/7	2.01 (1.00–4.05)	0.051	5/7	2.25 (1.19–4.27)	***0.013***
Duration of previous treatment						
<3 m	0/6	Reference		1/6	Reference	
≥3m	8/22	30.61 (0.28–33648)	0.338	9/22	2.87 (0.36–22.72)	0.317
Absolute neutrophil count						
<1 × 10^9/L	4/13	Reference		4/13	Reference	
≥1 × 10^9/L	4/15	0.83 (0.21-3.34)	0.792	6/15	1.28 (0.36-4.56)	0.700
Haemoglobin						
<54g/L	6/14	Reference		7/14	Reference	
≥54 g/L	2/14	0.30 (0.06–1.50)	0.143	3/14	0.37(0.10–1.44)	0.151
Platelet count						
<11 × 10^9/L	5/14	Reference		6/14	Reference	
≥11 × 10^9/L	3/14	0.59 (0.14–2.47)	0.470	4/14	0.65 (0.18–2.29)	0.499
Reticulocyte count						
<35 × 10^9/L	3/10	Reference		4/10	Reference	
≥35 × 10^9/L	5/18	0.88 (0.21–3.68)	0.859	6/18	0.79 (0.22–2.80)	0.716
Serum ferritin						
<1000ng/ml	3/16	Reference		5/16	Reference	
≧1000ng/ml	5/12	2.46 (0.59–10.32)	0.219	5/12	1.46 (0.42–5.06)	0.551
Number of transfused PRCs						
<78.75U	2/17	Reference		3/17	Reference	
≥78.75U	6/11	5.76(1.16–28.69)	***0.032***	7/11	5.08 (1.30–19.85)	***0.019***
Lymphocyte percentage						
<60%	3/12	Reference		4/12	Reference	
≥60%	5/16	1.37(0.33–5.75)	0.668	6/16	1.24 (0.35–4.39)	0.743
CD4(+)/CD8(+) T cell						
<1.1	4/10	Reference		4/10	Reference	
≥1.1	4/18	0.49(0.12–1.94)	0.307	6/18	0.75 (0.21–2.64)	0.649

#### Survival comparison based on DD differences

**Figure 3.**
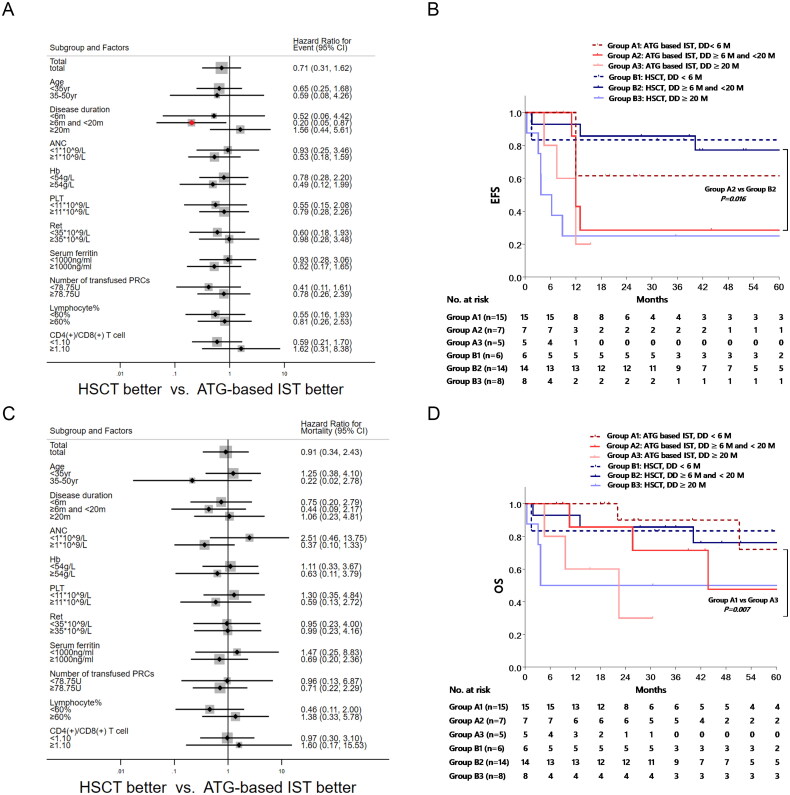
Risk analysis and the comparison of OS and EFS based on disease duration. The hazard ratio for events (3A) and mortality (3C) was estimated based on age, disease duration (DD), baseline absolute neutrophil count (ANC), haemoglobin (HB), platelet count (PLT), serum ferritin (SF), number of transfused packed red cells (PRCs), lymphocyte percentage, and CD4/CD8 ratio between ATG-based IST group and HSCT group by Cox regression. Patients with DD of 6 to 20 months in HSCT group had a lower risk of events than IST group (*p* = 0.032). The EFS (3B) and OS (3D) of different treatment regime based on DD were compared by Kaplan-Meier method. Patients with shorter DD had a significantly better EFS, and the HSCT group with a DD of 6 to 20 months showed a better EFS when compared to ATG based IST group (*p* = 0.016).

We initially assessed the influence of different treatment approaches on survival outcomes, taking into account factors such as age, disease duration (DD), previous treatment duration, baseline absolute neutrophil count (ANC), haemoglobin (Hb) level, platelet count (PLT), reticulocyte count (Ret), serum ferritin (SF), lymphocyte percentage, and CD4/CD8 ratio. Subgroup analysis unveiled that DD ranging from 6 to 20 months was the sole factor linked to heightened events in the ATG-based IST group, compared to the HSCT group (hazard ratio [HR] = 0.20, 95% confidence interval [CI]: 0.05–0.87, *p* = 0.032) (Figure 3(A)).

The Kaplan–Meier method was employed to juxtapose the EFS with regard to distinct treatment regimens and DD, with the objective of illuminating the influence of DD on the optimization of treatment strategies. Within the ATG-based IST group, the 5-year EFS rates for patients with DD ≤6 months, 6–20 months, and ≥20 months stood at 61.5% ± 13.5%, 28.6% ± 17.1%, and 20.0% ± 17.9%, respectively. Remarkably, patients with DD ≤6 months displayed a superior EFS compared to those with DD ≥20 months (*p* = 0.025). In the HSCT group, the corresponding rates were 83.3% ± 15.2%, 77.1% ± 11.7%, and 25.0% ± 15.3%, respectively. Importantly, patients within the 6–20 months DD range showcased a significantly improved EFS than those with DD ≥20 months (*p* = 0.004). Noteworthy is the finding that HSCT demonstrated heightened EFS exclusively in the ATG-based IST group for patients with DD within the 6-20 months bracket (77.1% ± 11.7% vs. 28.6% ± 17.1%, *p* = 0.016) (Figure 3(B)). Conversely, no statistically significant disparities were detected in OS across the pre-defined patient subgroups (Figure 3(C)), and the Kaplan–Meier analysis divulged dissimilarities in OS solely within the ATG-based IST group concerning DD ranging from ≤6 months to ≥20 months (72.0% ± 17.8% vs. 30.0% ± 23.9%, *p* = 0.007) (Figure 3(D)).

### Events and causes of death

Frequency and causes of events and deaths are presented in Supplemental Table 2. Within the ATG-based IST group (*n* = 27), 25 events transpired, while the HSCT group (*n* = 28) encountered 11 events (*p* = 0.005). Among the 27 patients subjected to ATG-based IST, five patients encountered a solitary event, seven patients experienced two events, and two patients suffered three events. In this group, 11 patients exhibited treatment failure within a 12-month observation period, wherein two progressed to SAA and one developed acute leukemia. Three of these patients underwent immediate HSCT, yet two succumbed during the early transplant stages. In the HSCT cohort, eight patients grappled with Epstein–Barr virus (EBV) reactivation, with one individual being diagnosed with post-transplant lymphoproliferative disorder (PTLD) following lymph node biopsy conducted 9 months post-HSCT. Notably, no patient within our cohort experienced primary or secondary graft failure. Furthermore, six months after HSCT, a patient with a 74-month DD showcased an expansion of a paroxysmal nocturnal haemoglobinuria (PNH) clone (surging from 3.7% to 21.5%), yet managed to attain sustained hematologic complete remission during an 80-month follow-up period. In the ATG-based IST group, two cases also demonstrated PNH clone expansion (from 2.2% to 11% and 0.9% to 13.3%) over follow-up durations of 19 and 60 months, respectively. Moreover, the patient harbouring a notable 39.2% PNH clone before treatment witnessed a decline to 0.3% at the 16-month mark after ATG-based IST. For the remaining patients in both groups, PNH clones remained stable (<5%) or exhibited a marginal decrease post-treatment. While there existed no significant variance in the count of deaths between the two groups, notable distinctions emerged in the causes of death (*p* = 0.007). The HSCT group predominantly succumbed to treatment-related factors (75%) within 100 days post-HSCT, encompassing infections, thrombotic microangiopathy, and heart failure. Conversely, the ATG-based IST group’s primary causes of death encompassed disease-related pneumonia, major bleeding, and disease progression.

### Complications

Within the HSCT group, the cumulative incidence of grade II–IV acute graft-versus-host disease (aGVHD) and grade III–IV aGVHD at 100 days post-transplantation stood at 25% (7/28) and 3.57% (1/28), respectively. Predominantly, the skin and intestines were the major organs affected by aGVHD. Moreover, the five-year cumulative incidence of chronic graft-versus-host disease (cGVHD) reached 39.3% (11/28). It is notable that no significant disparities were observed in the incidence of aGVHD and cGVHD across various graft types (Supplemental Figure 3A and 3B). In the ATG-based IST group, the incidence of serum sickness reaction tallied at 37.0% (10/27).

## Discussion

In China, the initial therapeutic approach recommended for TD-NSAA was centred on CsA-based IST. Nevertheless, close to half of the patients exhibited a lack of response to this intervention or underwent relapse or progression during the course of therapy [14]. In the light of this, there exists a dearth of extensive data concerning subsequent therapeutic choices for patients experiencing non-response to CsA-based IST, whether that involves transitioning to ATG-based IST or opting for HSCT. This study was meticulously designed to systematically assess and compare the efficacy and safety profiles of ATG-based IST and HSCT in the context of treating TD-NSAA. The overarching aim is to provide a definitive optimal choice tailored for this specific patient population.

Song et al. [14] reported a noteworthy short-term response rate (83.9% at 6 months) in TD-NSAA patients treated with ATG-based IST. However, our retrospective study revealed a comparatively lower response rate of 44.4% after 6 months, which only marginally increased to 48.1% after 12 months for patients undergoing ATG-based IST. This discrepancy in efficacy could be attributed to disparities in baseline characteristics. Notably, while all TD-NSAA patients in Song’s study received frontline ATG-based IST, almost half of our study cohort had previously undergone CsA treatment for more than three months before embarking on ATG-based IST. This subgroup, characterized by non-responsiveness to treatment, exhibited a notably poorer five-year EFS compared to those who received frontline treatment for TD-NSAA. With the growing prevalence of haploidentical transplantation in China, a substantial proportion of TD-NSAA patients now have access to donors. In this context, HSCT, irrespective of prior therapeutic history, demonstrated higher response rates and accelerated hematopoietic recovery in contrast to ATG-based IST—a finding consistently aligned with observations in studies concerning SAA [15, 16]. Despite these disparities, our study did not yield significant differences in EFS and OS between the ATG-based IST and HSCT subgroups.

Cox analysis was undertaken, based on treatment regimens, to unearth prognostic factors. It was found that a transfused RRCs count ≥78.75 U emerged as the sole independent risk factor for OS within the ATG-based IST group. Notably, a higher transfusion burden has been correlated with decreased overall survival and heightened non-relapse mortality in MDS patients undergoing HSCT. This is primarily attributed to pre-transplant transfusion history and consequent secondary iron overload. The accumulation of iron impedes the bone marrow’s hematopoietic precursor cells, particularly immature erythroblasts, by augmenting reactive oxygen species and quelling the expression of B-cell lymphoma-2, thereby fostering the apoptosis of immature erythroblasts and heightening dependence on erythrocyte transfusion support [17, 18]. Importantly, iron overload has also been linked to less favorable outcomes in SAA patients undergoing IST [19]. In the HSCT group, a prolonged DD (≥20 months) and a heightened number of transfused RRCs (≥78.75 U) were identified as adverse factors influencing EFS. Prolonged reliance on blood transfusion, stemming from extended DD, engenders elevated iron accumulation. Though HSCT can be a beneficial strategy for AA patients with cytogenetic anomalies, the achievement of HSCT is constricted by dysfunctions within the hematopoietic microenvironment, notably iron overload [20–22]. These observations underscore the critical role of iron chelation therapy as a prelude to treatment for TD-NSAA patients harbouring iron overload. Furthermore, this study pinpointed the most suitable treatment avenue for distinct patient cohorts. For TD-NSAA patients with a DD spanning 6 to 20 months, HSCT exhibited superior EFS rates compared to ATG-based IST. Within the group of patients manifesting a DD under 6 months, both HSCT and ATG-based IST demonstrated comparable survival outcomes. However, for patients grappling with a DD exceeding 20 months, neither therapeutic option yielded a favourable prognosis, underscoring the urgency for swift initiation of frontline treatment.

Complications and early mortality present formidable hurdles in the landscape of HSCT. Notably, six out of eight deaths associated with transplantation transpired within the initial 100 days post stem cell infusion. Remarkably, patients burdened with iron overload exhibited a higher mortality rate relative to those unencumbered by this condition. Iron overload not only precipitates deleterious impacts on vital organs such as the heart, liver, and pancreas, but it also augments susceptibility to infections [23]. Intriguingly, the incidence of aGVHD and cGVHD demonstrated no significant variance between matched donor and alternative donor (AD) transplantation, thereby underscoring the viability of employing haploidentical HSCT within our patient cohort.

In summation, this study underscores the paramount importance of vigilant monitoring and proactive management of TD-NSAA patients. The consideration of antecedent treatment status, duration of disease, quantification of transfused RRCs, and assessment of SF levels emerges as pivotal in steering the decision-making process between ATG-based IST and allo-HSCT for the subset of patients grappling with TD-NSAA. Mostly, allo-HSCT achieved a faster and higher response than ATG-based IST, and a better EFS also observed in HSCT group if patients with a disease duration spanning 6 to 20 months. More prospective studies are needed to consolidate these findings.

## Supplementary Material

Supplemental Material

## Data Availability

The data used and/or analysed during the current study are available from the corresponding author upon a reasonable request.
