# Supplementary material for: Comparison of anti-thymocyte globulin-based immunosuppressive therapy and allogeneic hematopoietic stem cell transplantation in patients with transfusion-dependent non-severe aplastic anaemia: a retrospective study from a single centre
Source: Ann Med. 2023 Oct 23;55(2):2271475. doi: 10.1080/07853890.2023.2271475 (PMC10595398; doi:10.1080/07853890.2023.2271475)
Supplement: Supplemental Material [file IANN_A_2271475_SM4491.zip › Supplelmental Tables.docx]

**Supplemental Table 1. Clinical features of 28 patients in the HSCT group**

| Variable | N=28 |
| --- | --- |
| Donor median age/ years (range) | 32 (13-54) |
| Type of donor/ n (%) |  |
| MSD  UD  HID | 8 (28.6%)  7 (25.0%)  13 (46.4%) |
| Donor-recipient relationship/ n (%)  Haplo (patient-child)  Haplo (child-patient)  Siblings  Unrelated | 5 (17.9%)  2 (7.1%)  14 (50.0%)  7 (25.0%) |
| Donor-recipient sex match/ n (%) |  |
| Male-male | 7 (25%) |
| Male-female | 10 (35.7%) |
| Female-male | 4 (14.3%) |
| Female-female | 7 (25%) |
| Blood types of donor to recipient/ n (%) |  |
| Matched | 18 (64.3%) |
| Major mismatched | 6 (21.4%) |
| Minor mismatched | 4 (14.3) |
| Major and minor mismatched | 0 |
| Source of graft/ n (%)  PB  BM+PB | 7 (25%)  21 (75%) |
| HLA-matched/ n (%)  5/10  6/10  7/10  8/10  9/10  10/10 | 6 (21.4%)  3 (10.7%)  3 (10.7%)  1 (3.6%)  6 (21.4%)  9 (32.1%) |
| Conditioning regimen  FLU/CY/ATG  BU/CY/ATG  BU/CY | 24 (85.7%)  2 (7.1%)  2 (7.1%) |
| MNC(×10^8^/kg )/ Median (range)  CD34+ cell(×10^6^/kg )/ Median (range) | 6.75 (3.56-14.58)  5.52 (3.50-8.83) |

**Note:** MSD: matched sibling donor; UD: unrelated donor; HID: haplo-identical donor; BM: bone marrow; PB: peripheral blood; HLA: human leucocyte antigen; FLU: Fludarabine; CY: Cyclophosphamide; ATG: antithymocyte globulin; BU: Busulfan; MNC: mononuclear cell.

**Supplemental Table 2. Events and causes of death in ATG based IST group and HSCT group**

|  | ATG-based IST(n=27) | HSCT (n=28) | *P* |
| --- | --- | --- | --- |
| Patients with an event  With a single event  With two events  With three events | 14  5(18.5%)  7(25.9%)  2(7.4%) | 10  9(32.1%)  1(3.6%)  0 | 0.282 |
| Number/type of events  Progression to severe aplastic anemia  Transformed into acute leukemia  PNH  PTLD  Death  12 months non-response | 25  2(7.4%)  1(3.7%)  0  0  8(29.6%)  11(40.7%) | 11  0  0  1(3.6%)  1(3.6%)  8(28.6%)  0 | ***0.005*** |
| Grade III–IV aGVHD  Conversion treatment | 0  3(11.1%) | 1(3.6%) |  |
| Number/type of deaths  Early deaths  Infection  Thrombotic Microangiopathy  Heart failure  Disease-related deaths  Bleeding  Infection  Event-related deaths  Leukemia  Progression | 8(29.6%)  0  0  0  0  6(75%)  3  3  2(25%)  1  1 | 8(28.6%)  6(75%)  3  1  2  2(25%)  0  2  0  0  0 | ***0.007***  0.206 |

**Note:** PNH: paroxysmal nocturnal hemoglobinuria; PTLD: post-transplant lymphoproliferative disorder.

.
