# Supplementary material for: Comparison of anti-thymocyte globulin-based immunosuppressive therapy and allogeneic hematopoietic stem cell transplantation in patients with transfusion-dependent non-severe aplastic anaemia: a retrospective study from a single centre
Source: Ann Med. 2023 Oct 23;55(2):2271475. doi: 10.1080/07853890.2023.2271475 (PMC10595398; doi:10.1080/07853890.2023.2271475)
Supplement: Supplemental Material [file IANN_A_2271475_SM4491.zip › Supplemental Figure legend.docx]

**Supplemental Figure Legend:**

**Supple Figure 1. Flow chart of the patients included in this study.** 97 patients were assessed between September 2011 and December 2019 in our center, and finally 55 patient who treated with HSCT or ATG-based IST were enrolled for the further efficacy and safety analysis.

**Supple Figure 2.** ROC curve for disease duration and number of transfused packed red cells at overall survival (OS). Disease duration (DD) was found to have 66.7% sensitivity and 83.3% specificity to predict the OS at a cutoff DD of 6 months in ATG-based IST (2A), while 54.5% sensitivity and 94.1% specificity at a cutoff DD of 20 months in HSCT (2B). Number of transfused packed red cells (PRCs) was found to have 75% sensitivity and 82.1% specificity to predict the OS at a cutoff number of transfused (PRCs) of 78.75U (2C). With P < .05 considered statistically significant. AUC: Areas under the curve; ROC: Receiver operating characteristic.

**Supple Figure 3. Cumulative incidence of II-IV acute GVHD and moderate/severe chronic GVHD comparison based on donor sources**. The cumulative incidence of acute GVHD (3A) and chronic GVHD (3B) were compared in HSCT group with different donor type (MUD vs AD), and no statistic differences were observed. MUD: matched unrelated donor; AD: alternative donor; GVHD: graft-versus-host disease.
