# Supplementary figures and images for: Comparison of anti-thymocyte globulin-based immunosuppressive therapy and allogeneic hematopoietic stem cell transplantation in patients with transfusion-dependent non-severe aplastic anaemia: a retrospective study from a single centre
Source: Ann Med. 2023 Oct 23;55(2):2271475. doi: 10.1080/07853890.2023.2271475 (PMC10595398; doi:10.1080/07853890.2023.2271475)

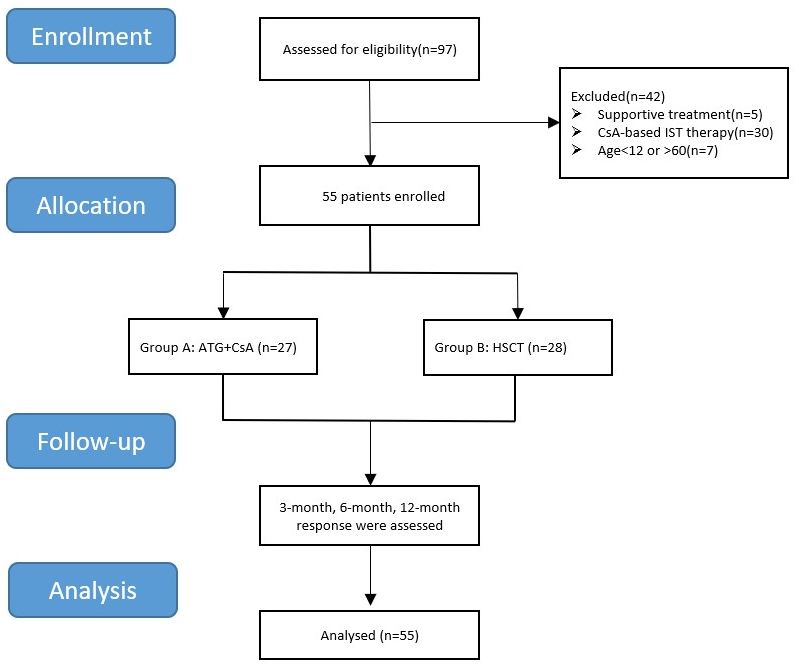

Supplement: Supplemental Material [file IANN_A_2271475_SM4491.zip › Supplemental Fig.1.tiff]

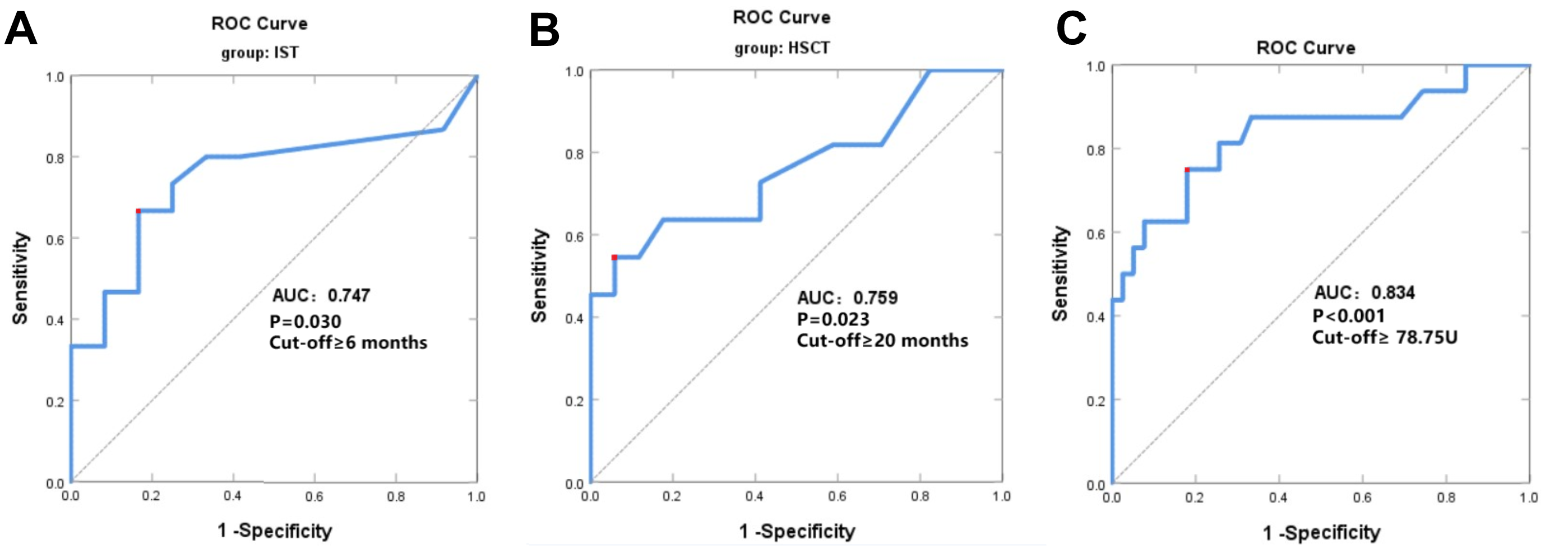

Supplement: Supplemental Material [file IANN_A_2271475_SM4491.zip › Supplemental Fig.2.tif]

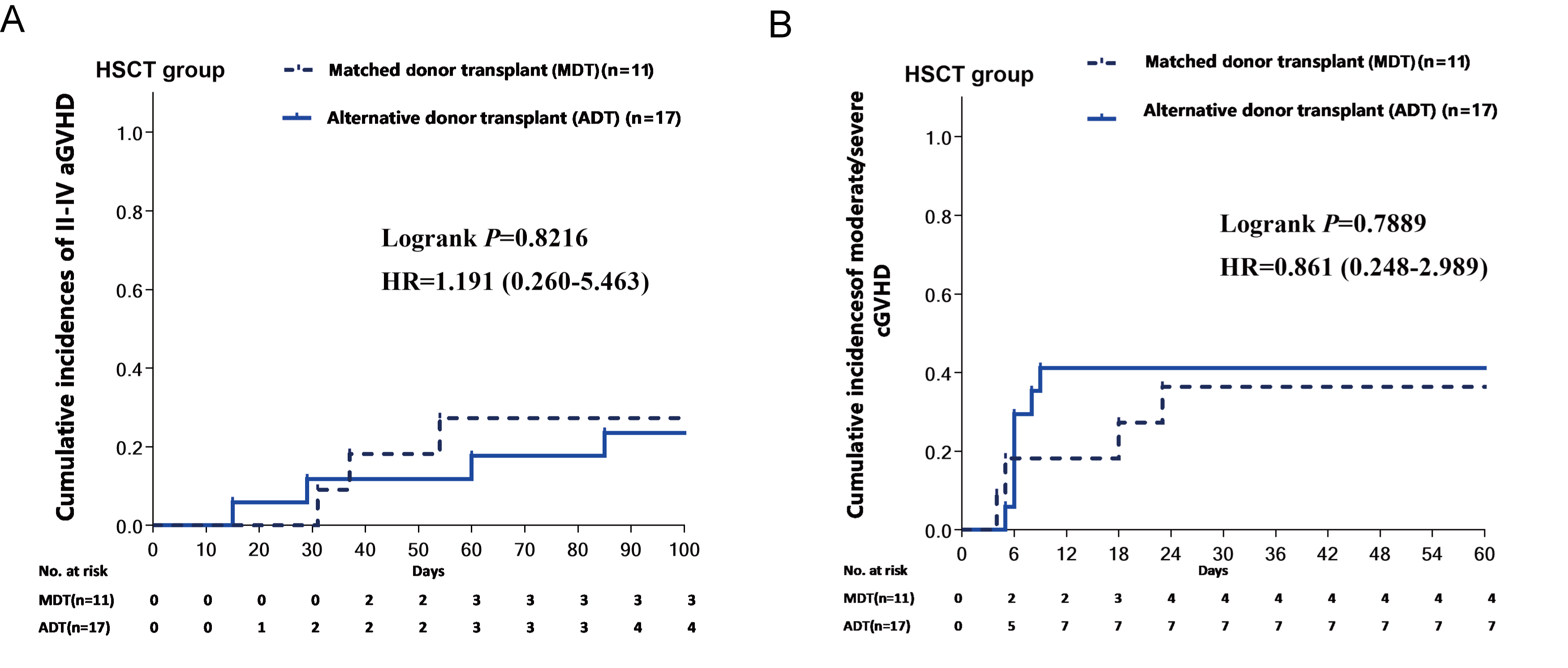

Supplement: Supplemental Material [file IANN_A_2271475_SM4491.zip › Supplemental Fig.3.tif]
